# Supplementary material for: Searching large-scale scRNA-seq databases via unbiased cell embedding with Cell BLAST
Source: Nat Commun. 2020 Jul 10;11:3458. doi: 10.1038/s41467-020-17281-7 (PMC7351785; doi:10.1038/s41467-020-17281-7)
Supplement: Supplementary file 1 — Supplementary Information [file 41467_2020_17281_MOESM1_ESM.pdf]

Supplementary Information

Searching large-scale scRNA-seq databases via unbiased cell embedding with Cell BLAST

Cao et al.

Method	Capabilities		
	**Dimension reduction**	**Batch correction**	**Cell querying**
PCA[1]	Yes	No	No
ZIFA[2]	Yes	No	No
ZINB WaVE[3]	Yes	Yes, but not tested [1]	No
Dhaka[4]	Yes	No	No
DCA[5]	Yes	No	No
tSNE[6]	Yes	No	No
UMAP[7]	Yes	No	No
scVI[8]	Yes	Yes	No
SAUCIE[9]	Yes	Yes	No
scPhere[10]	Yes	Yes	No
scScope[11]	Yes	Yes, but not tested [2]	No
ComBat[12]	No	Yes	No
MNN[13]	No	Yes	No
CCA[14]	No	Yes	No
CCA anchor[15]	No	Yes	No [3]
Harmony[16]	No	Yes	No
scmap[17]	No	No	Yes
CellFishing.jl[18]	No	No	Yes
Cell BLAST	Yes	Yes	Yes

Supplementary Table 1 Benchmarked methods and their capabilities.
[1, 2] Though ZINB-WaVE and scScope do support simple linear batch effect correction, they are not state-of-the-art batch effect correction methods so we did not compare their performance here.
[3] Though CCA anchor supports label transfer based on mutual nearest neighbor anchors, it is conceptually different from cell querying discussed in this paper. Key differences include: (i) it is not possible to transfer label for single query cells using CCA anchor because mutual nearest neighbors would be ill-defined. (ii) CCA anchor does not reject query cells at all, and does not provide a distance/similarity metric which can be used to filter irrelevant cells, thus querying specificity is always zero.

Dataset Name	Organism	Organ Profiled	Experimental Platform	Number of cells	Number of selected genes	Used In
Guo[19]	Human	Testis	10x[20]	6,490	1,000	DR
Muraro[21]		Pancreas	CEL-Seq2[22]	2,122	990	DR, BC
Xin_2016[23]			SMARTer[24]	609	643	BC
Lawlor[25]			SMARTer[24]	370	848	BC
Segerstolpe[26]			Smart-seq2[27]	1,070	967	BC
Enge[28]			Smart-seq2[27]	2,282	350	BC
Baron_human[29]			inDrop[30]	8,569	769	DR, BC
Baron_mouse[29]	Mouse		inDrop[30]	1,886	419	BC
Adam[31]		Kidney	Drop-seq[32]	3,660	859	DR
Plasschaert[33]		Trachea	inDrop[30]	6,977	960	DR, BC
Montoro_10x[34]			10x[20]	7,193	458	BC
Macosko[32]		Retina	Drop-seq[32]	44,808	727	DR
Bach[35]		Mammary Gland	10x[20]	23,184	694	DR
Quake_Smart-seq2[36]		20 Organs	Smart-seq2[27]	44,949	1,026	BC
Quake_10x[36]		12 Organs	10x[20]	55,656	965	BC

Supplementary Table 2 Datasets used in dimensionality reduction and batch effect correction benchmarking. DR, dimension reduction benchmarking; BC, batch effect correction benchmarking.

Group	Role	Dataset Name	Organism	Organ Profiled	Experimental Platform
Pancreas	Reference	Baron_human[29]	Human	Pancreas	inDrop[30]
Pancreas	Reference	Xin_2016[23]	Human	Pancreas	SMARTer[24]
Pancreas	Reference	Lawlor[25]	Human	Pancreas	SMARTer[24]
Pancreas	Positive query	Muraro[21]	Human	Pancreas	CEL-Seq2[22]
Pancreas	Positive query	Segerstolpe[26]	Human	Pancreas	Smart-seq2[27]
Pancreas	Positive query	Enge[28]	Human	Pancreas	Smart-seq2[27]
Pancreas	Negative query	Wu_human[37]	Human	Kidney	10x[20]
Pancreas	Negative query	Zheng[20]	Human	PBMC	10x[20]
Pancreas	Negative query	Philippeos[38]	Human	Skin	Smart-seq2[27]
Trachea	Reference	Montoro_10x[34]	Mouse	Trachea	10x[20]
Trachea	Positive query	Plasschaert[33]	Mouse	Trachea	inDrop[30]
Trachea	Negative query	Baron_mouse[29]	Mouse	Pancreas	inDrop[30]
Trachea	Negative query	Park[39]	Mouse	Kidney	10x[20]
Trachea	Negative query	Bach[35]	Mouse	Mammary Gland	10x[20]
Trachea	Negative query	Macosko[32]	Mouse	Retina	Drop-seq[32]
Mammary Gland	Reference	Bach[35]	Mouse	Mammary Gland	10x[20]
Mammary Gland	Positive query	Giraddi_10x[40]	Mouse	Mammary Gland	10x[20]
Mammary Gland	Positive query	Quake_Smart-seq2_ Mammary_Gland[36]	Mouse	Mammary Gland	Smart-seq2[27]
Mammary Gland	Positive query	Quake_10x_ Mammary_Gland[36]	Mouse	Mammary Gland	10x[20]
Mammary Gland	Negative query	Baron_mouse[29]	Mouse	Pancreas	inDrop[30]
Mammary Gland	Negative query	Park[39]	Mouse	Kidney	10x[20]
Mammary Gland	Negative query	Plasschaert[33]	Mouse	Trachea	inDrop[30]
Mammary Gland	Negative query	Macosko[32]	Mouse	Retina	Drop-seq[32]

Lung	Reference	Quake_10x_ Lung[36]	Mouse	Lung	10x[20]
	Positive query	Quake-Smart-seq2_ Lung[36]			Smart-seq2[27]
	Negative query	Baron_mouse[29]		Pancreas	inDrop[30]
		Park[39]		Kidney	10x[20]
		Bach[35]		Mammary Gland	10x[20]
		Plasschaert[33]		Trachea	inDrop[30]

Supplementary Table 3 Datasets used in benchmarking query-based cell typing.

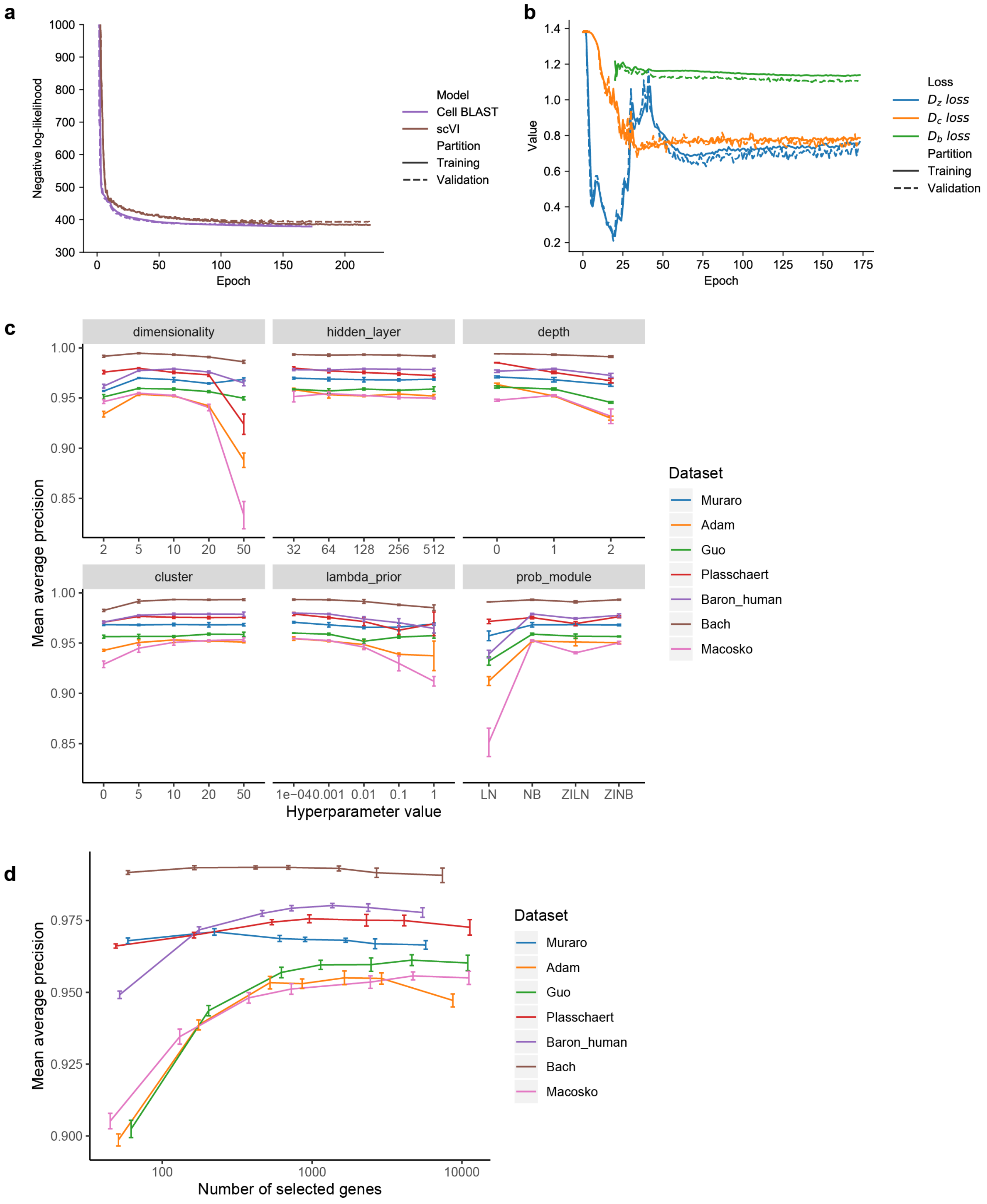

a
Negative log-likelihood
Epoch
Model
Cell BLAST
scVI
Partition
Training
Validation
b
Value
Epoch
Loss
D_z loss
D_c loss
D_b loss
Partition
Training
Validation
c
dimensionality
hidden_layer
depth
cluster
lambda_prior
prob_module
Mean average precision
Hyperparameter value
LN
NB
ZILN
ZINB
Dataset
Muraro
Adam
Guo
Plasschaert
Baron_human
Bach
Macosko
d
Mean average precision
Number of selected genes
Dataset
Muraro
Adam
Guo
Plasschaert
Baron_human
Bach
Macosko

Supplementary Figure 1 Training dynamics and hyperparameter test. (**a**) Negative log-likelihood of Cell BLAST converges fast and stably, with similar dynamics as scVI. (**b**) Adversarially trained discriminator losses in Cell BLAST also converge stably. Note that there is a 20-epoch burn-in stage during which adversarial batch alignment is not enabled, allowing model to fit all data variations first. The batch discriminator $D_{\mathbf{b}}$ only starts training after the burn-in stage. (**c**) Mean average precision under different hyperparameter settings in each benchmark dataset across different random initializations (n = 16). Hyperparameters "dimensionality" is the dimensionality D of cell embedding **l**, "hidden_layer" is the dimensionality of hidden layers in the encoder, decoder and discriminators, "depth" is the number of hidden layers in encoder, decoder and discriminators, "cluster" is the number of categories K in latent variable **c**, "lambda_prior" is the regularization strength $\lambda_{\mathbf{z}}$ and $\lambda_{\mathbf{c}}$ for prior matching, "prob_module" is the generative distribution used in the model, where "LN" represents log normal, "NB" represents negative binomial, "ZILN" represents zero-inflated log normal, and "ZINB" represents zero-inflated negative binomial. (**d**) Mean average precision of Cell BLAST (with the default hyperparameter setting) under different-sized feature sets in each benchmark dataset across different random initializations (n = 16). Error bars indicate mean ± s.d.

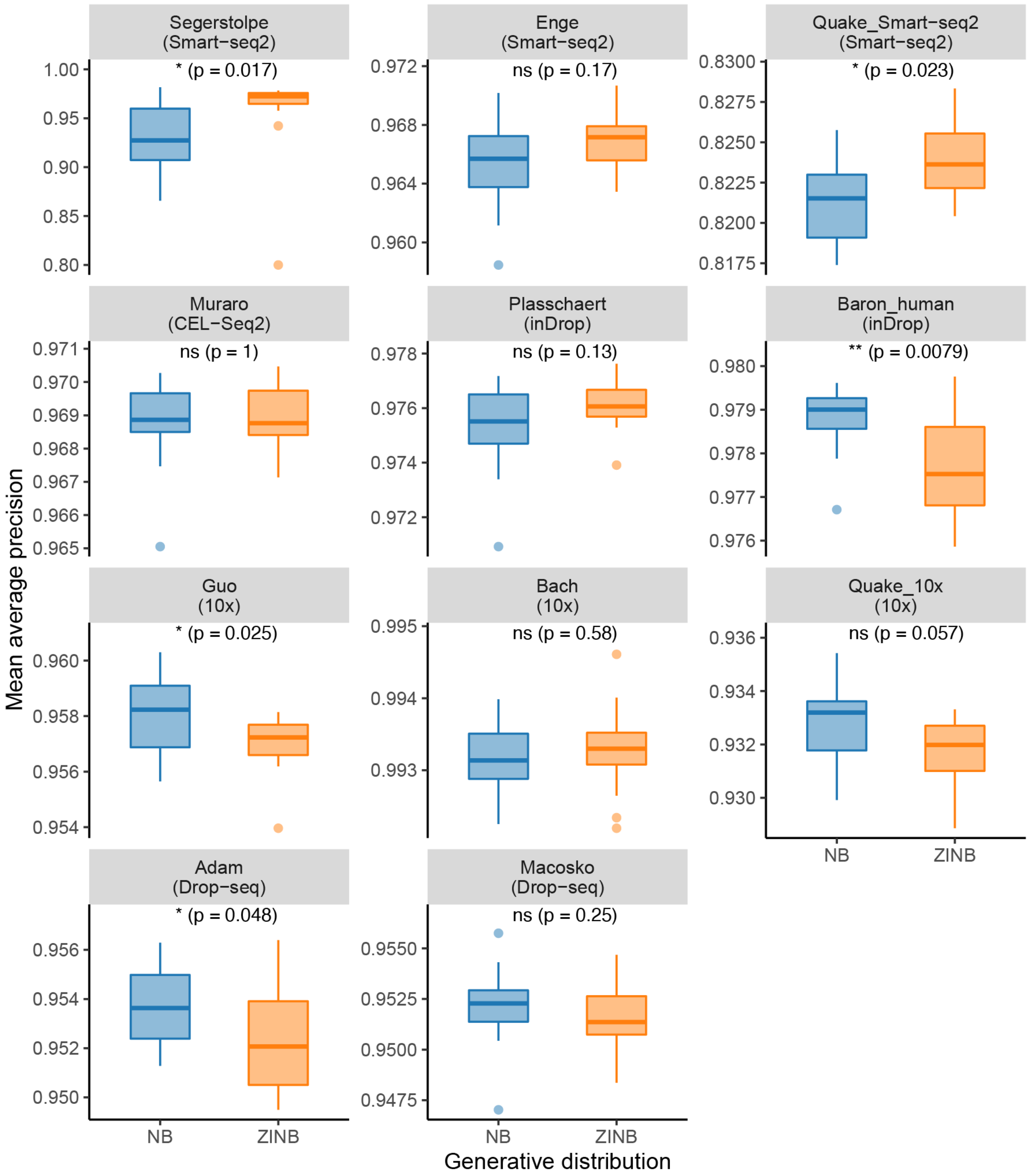

Supplementary Figure 2 Comparing models employing standard vs zero-inflated negative binomial as generative distribution, trained on data from different experimental protocols, across different random initializations (n = 16). Box plots indicate the median (center lines), 1st and 3rd quartiles (hinges), minimal and maximal point within 1.5 times the interquartile range starting from the hinges (whiskers). Statistical significance is evaluated by two-sided Wilcoxon rank-sum test. Significance indicators "**" indicates $P < 0.01$, "*" indicates $P < 0.05$, and "ns" indicates $P > 0.05$.

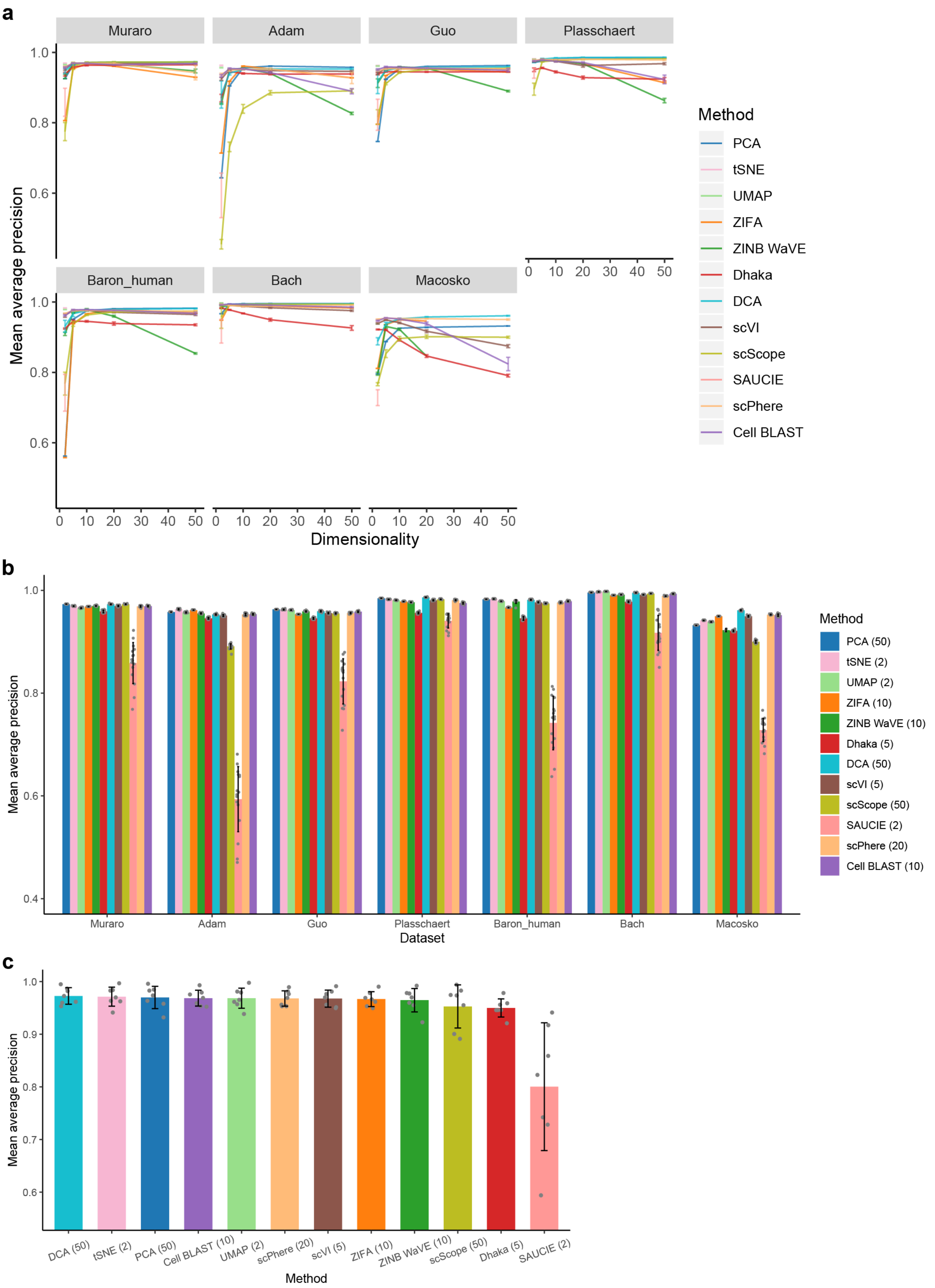

Supplementary Figure 3 Dimension reduction benchmark. (**a**) Mean average precision of benchmarked methods under different dimensionalities across different random initializations (n = 16). (**b**) Mean average precision of different dimensionality reduction methods in each of the seven benchmark datasets, obtained using their overall optimal dimensionalities (see brackets in the legend for optimal dimensionalities selected), across different random initializations (n = 16). (**c**) Ranking methods by their mean average precision across different benchmark datasets (n = 7). Error bars indicate mean ± s.d.

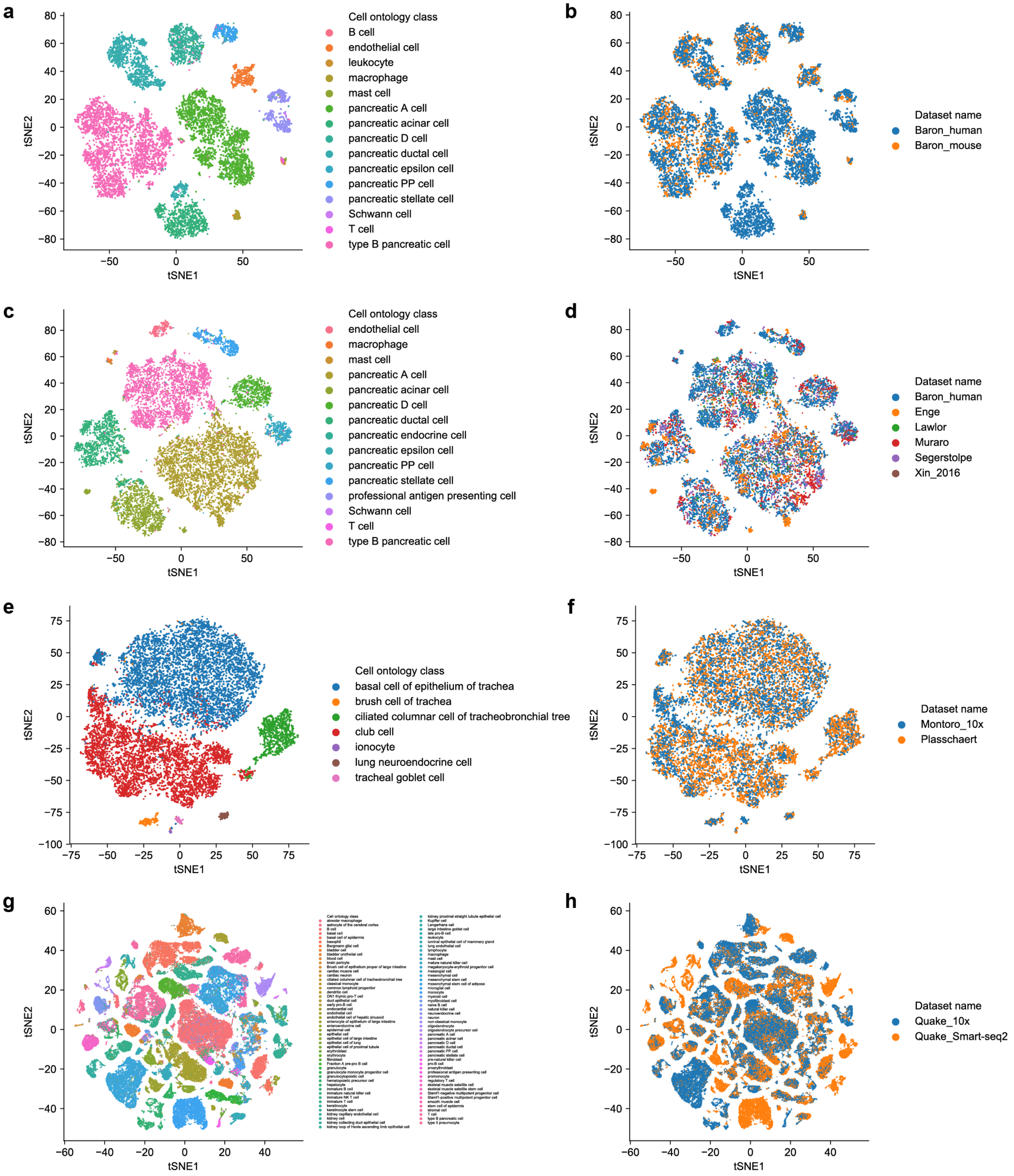

Supplementary Figure 4 t-SNE visualization of cell embeddings learned by our model on combinations of multiple datasets, with cross-dataset batch effect corrected. Figures in the left column are colored by cell type, while figures in the right column are colored by dataset. (**a-b**) “Baron_human”[29] and “Baron_mouse”[29]; (**c-d**) “Baron_human”[29], “Muraro”[21], “Enge”[28], “Segerstolpe”[26], “Xin_2016”[23] and “Lawlor”[25]; (**e-f**) “Montoro_10x”[34] and “Plasschaert”[33]; (**g-h**) “Quake_Smart-seq2”[36] and “Quake_10x”[36]. Notably, different batches are not always aligned perfectly, which is most evident in the (**h**). In these cases, cell type composition varies in different batches (see **Supplementary Data 1** for details), thus they are not supposed to be perfectly aligned *per se*.

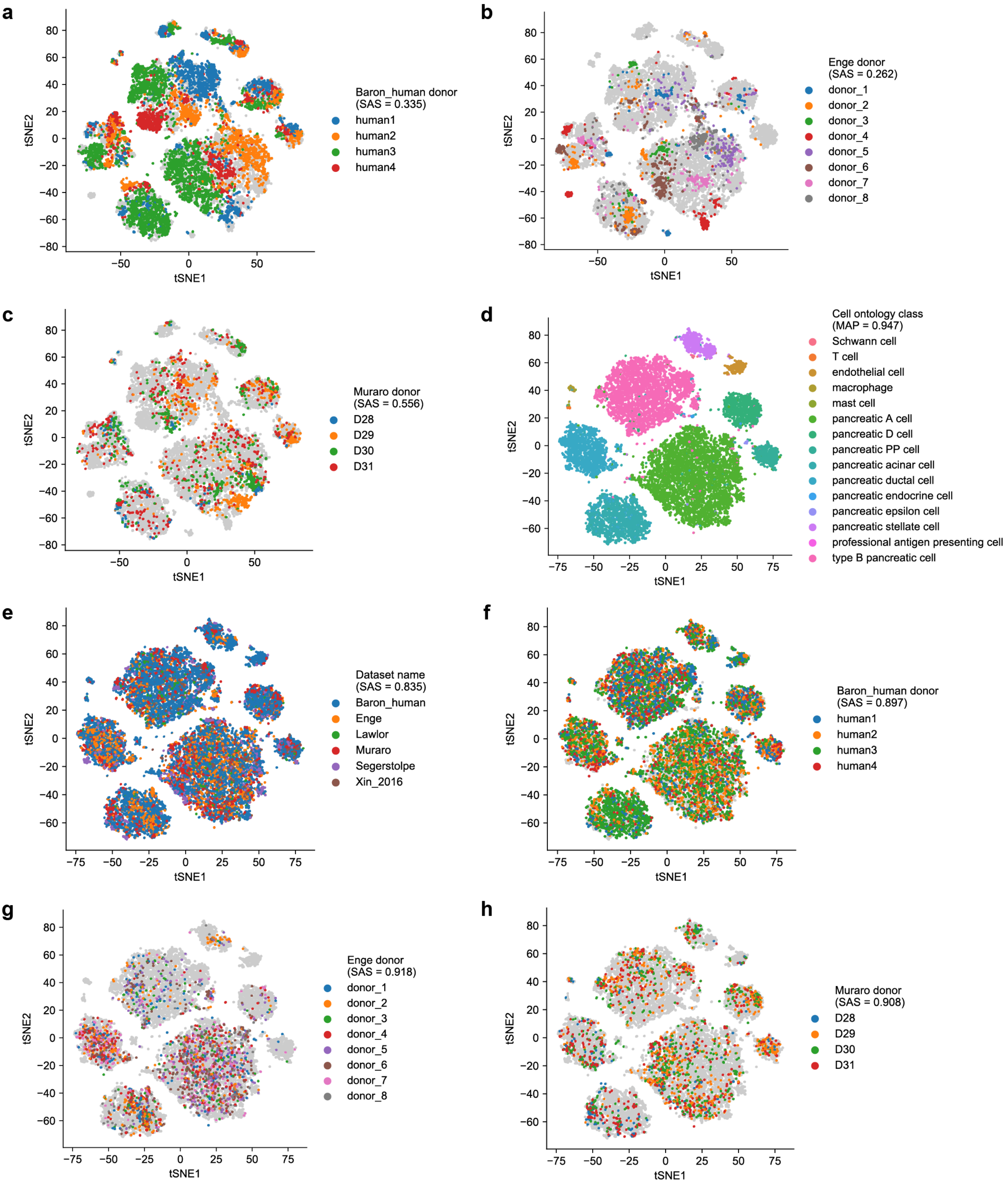
a
Baron_human donor
(SAS = 0.335)
human1
human2
human3
human4
b
Enge donor
(SAS = 0.262)
donor_1
donor_2
donor_3
donor_4
donor_5
donor_6
donor_7
donor_8
c
Muraro donor
(SAS = 0.556)
D28
D29
D30
D31
d
Cell ontology class
(MAP = 0.947)
Schwann cell
T cell
endothelial cell
macrophage
mast cell
pancreatic A cell
pancreatic D cell
pancreatic PP cell
pancreatic acinar cell
pancreatic ductal cell
pancreatic endocrine cell
pancreatic epsilon cell
pancreatic stellate cell
professional antigen presenting cell
type B pancreatic cell
e
Dataset name
(SAS = 0.835)
Baron_human
Enge
Lawlor
Muraro
Segerstolpe
Xin_2016
f
Baron_human donor
(SAS = 0.897)
human1
human2
human3
human4
g
Enge donor
(SAS = 0.918)
donor_1
donor_2
donor_3
donor_4
donor_5
donor_6
donor_7
donor_8
h
Muraro donor
(SAS = 0.908)
D28
D29
D30
D31
tSNE1
tSNE2

Supplementary Figure 5 Multilevel batch effect correction. The correction of inter-dataset batch effect does not automatically generalize to that within each dataset. Though the batch effect among six independent datasets are corrected (**Supplementary Fig. 4d**), different donors in each dataset remain separated (**a**-**c**). For such complex scenarios, our adversarial batch alignment can be easily extended with multiple batch discriminators, one for each source of batch effect (in this case four, including dataset of origin, donor in “Baron_human”, donor in “Enge”, and donor in “Muraro”), effectively correcting multiple levels of batch effect independently and simultaneously (**d**-**h**). (**a**-**c**) Cell embeddings learned with only cross-dataset batch effect correction, colored by (**a**) donor in “Baron_human”[29], (**b**) donor in “Enge”[28], (**c**) donor in “Muraro”[21]. (**d**-**h**) Cell embeddings learned with both cross-dataset and within-dataset batch effect correction, colored by (**d**) cell type, (**e**) dataset, (**f**) donor in “Baron_human”[29], (**g**) donor in “Enge”[28], (**h**) donor in “Muraro”[21]. Seurat alignment scores (SAS) for individual figures are shown under the legend titles.

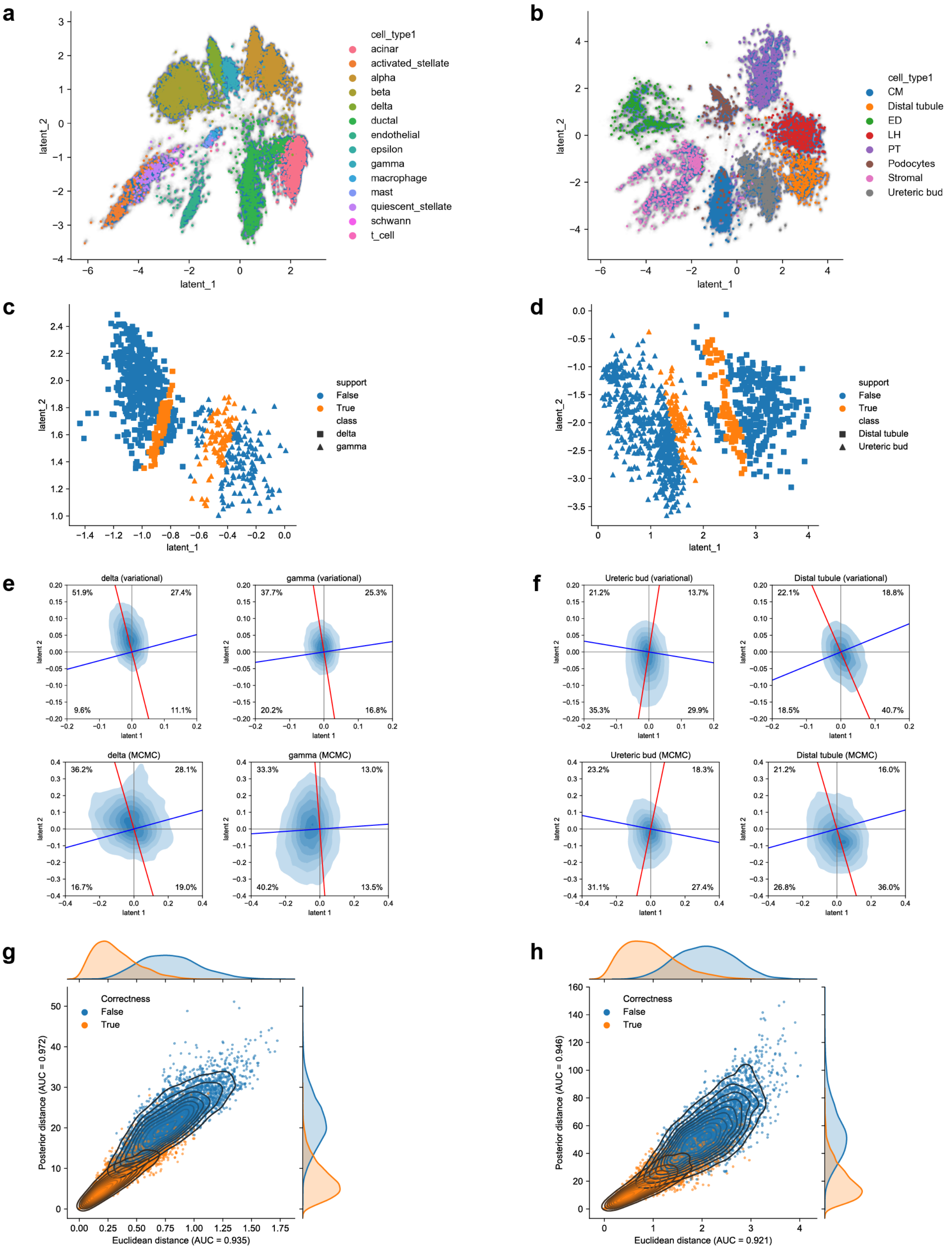
a
b
c
d
e
f
g
h
cell_type1
acinar
activated_stellate
alpha
beta
delta
ductal
endothelial
epsilon
gamma
macrophage
mast
quiescent_stellate
schwann
t_cell
CM
Distal tubule
ED
LH
PT
Podocytes
Stromal
Ureteric bud
latent_1
latent_2
support
False
True
class
delta
gamma
Distal tubule
Ureteric bud
delta (variational)
gamma (variational)
Ureteric bud (variational)
Distal tubule (variational)
delta (MCMC)
gamma (MCMC)
Ureteric bud (MCMC)
Distal tubule (MCMC)
latent 1
latent 2
51.9%
27.4%
9.6%
11.1%
37.7%
25.3%
20.2%
16.8%
21.2%
13.7%
35.3%
29.9%
22.1%
18.8%
18.5%
40.7%
36.2%
28.1%
16.7%
19.0%
33.3%
13.0%
40.2%
13.5%
23.2%
18.3%
31.1%
27.4%
21.2%
16.0%
26.8%
36.0%
Correctness
False
True
Posterior distance (AUC = 0.972)
Euclidean distance (AUC = 0.935)
Posterior distance (AUC = 0.946)
Euclidean distance (AUC = 0.921)

Supplementary Figure 6 Posterior visualization of Cell BLAST. (**a-d**) The commonly used Euclidean distance as cell-to-cell similarity metric may lead to artifact. To illustrate this, we visualized two-dimensional embeddings learned by Cell BLAST on the "Baron_human" and "Adam" dataset. E.g., delta cells and gamma cells in "Baron_human", as well as distal tubule cells and ureteric bud cells in "Adam" are relatively similar and always locate next to each other. Both of the cell clusters are elongated in a certain direction, such that cells near the border would be closer to cells of the opposite cell type than many cells of the same cell type in terms of Euclidean distance. Such phenomena can also be observed in many other cases. Cells embeddings (foreground) overlaid with posterior samples (blue cloud in the background) in 2-dimensional embedding space of the "Baron_human"[29] (**a**) and "Adam"[31] (**b**) dataset respectively. Cell type abbreviations in (**b**) represent cap mesenchyme (CM), endothelial cell (ED), loop of Henle (LH), and proximal tubule (PT). (**c**) Zoom-in view of the delta and gamma cell clusters in "Baron_human". (**d**) Zoom-in view of the distal tubule and ureteric bud cell clusters in "Adam". Cells near the cluster border highlighted in orange. (**e**, **f**) Aggregated posterior density of the border cells in "Baron_human" (**e**) and "Adam" (**f**). For both (**e**) and (**f**), panels in the first row are variational posterior densities, and panels in the second row are true posterior densities estimated via MCMC. Red and blue lines show the major axes of variation of the posterior distributions revealed by SVD. Variational posterior of Cell BLAST correctly captures the variation in the true posterior. (**g**, **h**) Comparison of Euclidean distance and variational posterior NPD in discerning cell pairs of the same cell type from cell pairs of different cell types, in "Baron_human" (**g**) and "Adam" (**h**) respectively.

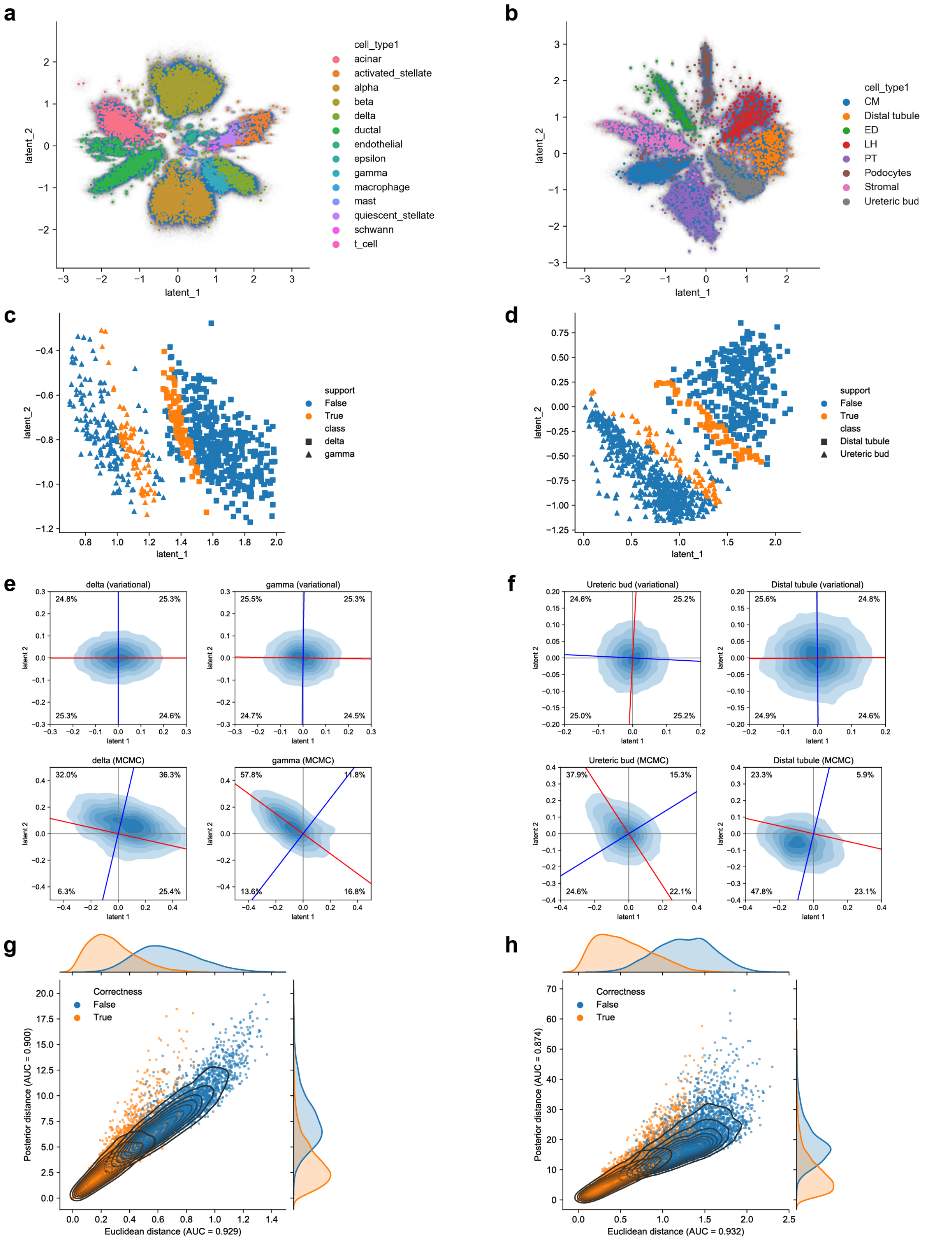

Supplementary Figure 7 Posterior visualization of scVI. (**a**, **b**) Cells embeddings (foreground) overlaid with posterior samples (blue cloud in the background) in 2-dimensional embedding space of the "Baron_human"[29] (**a**) and "Adam"[31] (**b**) dataset respectively. (**c**) Zoom-in view of the delta and gamma cell clusters in "Baron_human". (**c**) Zoom-in view of the distal tubule and ureteric bud cell clusters in "Adam". Cells near the cluster border highlighted in orange. (**e**, **f**) Aggregated posterior density of the border cells in "Baron_human" (**e**) and "Adam" (**f**). For both (**e**) and (**f**), panels in the first row are variational posterior densities, and panels in the second row are true posterior densities estimated via MCMC. (**g**, **h**) Comparison of Euclidean distance and variational posterior NPD in discerning cell pairs of the same cell type from cell pairs of different cell types, in "Baron_human" (**g**) and "Adam" (**h**) respectively.

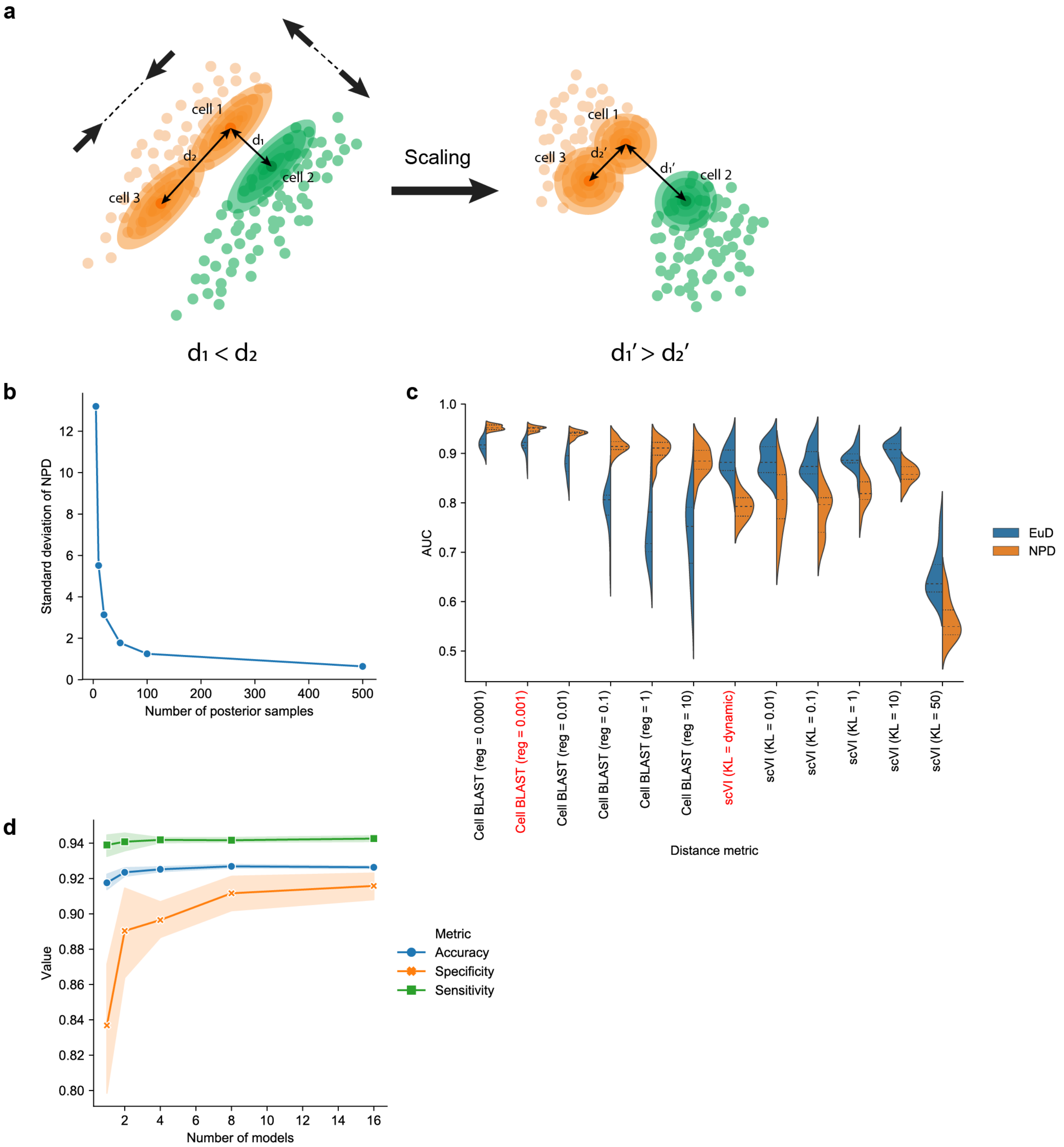

Supplementary Figure 8 Optimizing Cell BLAST querying strategy. (**a**) An illustration of how the variational posterior of Cell BLAST can inform the estimation of cell-to-cell similarity. Axes along which the posterior density is flatter indicate higher transcriptomic similarity compared to axes with steeply decreasing posterior density. So, distance along flatter axes should be compressed, while distance along steeper axes should be expanded. In this case the rescaled distance metric correctly assigns larger distance for cells of different types, while the original metric does not. (**b**) Standard deviation of posterior distance estimation decreases as the number of samples from the posterior distribution increases, saturating at 50 posterior samples with 10-dimensional cell embeddings. (**c**) AUC (Area Under Curve) of ROC curves for different distance metrics in distinguishing cross-dataset nearest neighbors of the same cell type and those of different cell types, all using 10-dimensional cell embeddings, across different random initializations (n = 16). EuD represents Euclidean distance. Horizontal lines inside the violins indicate quartiles. (**d**) Accuracy, specificity and sensitivity all increase as the number of models used for cell querying increases (n = 8 independent experiments), among which the improvement of specificity is the most significant. Error bars indicate mean ± s.d.

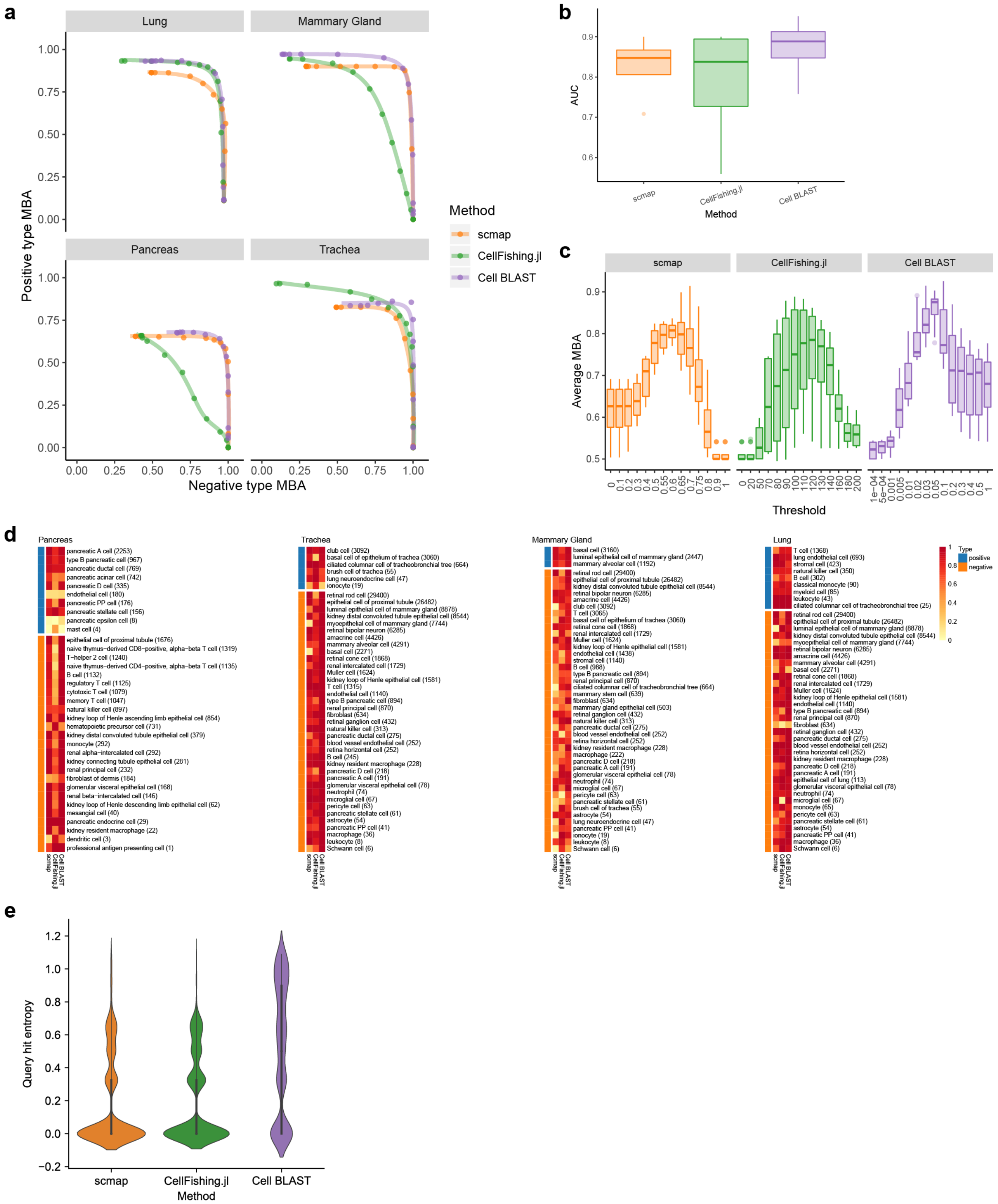

Supplementary Figure 9 Cell querying benchmark. (**a**) MBA curves reflecting the balance between predicting positive cell types and rejecting negative cell types. (**b**) AUC (Area Under Curve) of the MBA curves for each method across different query groups (n = 4). (**c**) Average MBA at different cutoff values (n = 16 experiments across four query groups for each method). (**d**) Heatmap showing cell type specific accuracies for each method. Numbers in brackets are population size of each cell type. (**e**) Shannon entropy of query hit distribution among three reference datasets in the pancreatic group. Higher values indicate that query hits are more evenly distributed among reference datasets, while lower values indicate that query hits are biased towards certain reference datasets. Box plots indicate the median (center lines), 1st and 3rd quartiles (hinges), minimal and maximal point within 1.5 times the interquartile range starting from the hinges (whiskers).

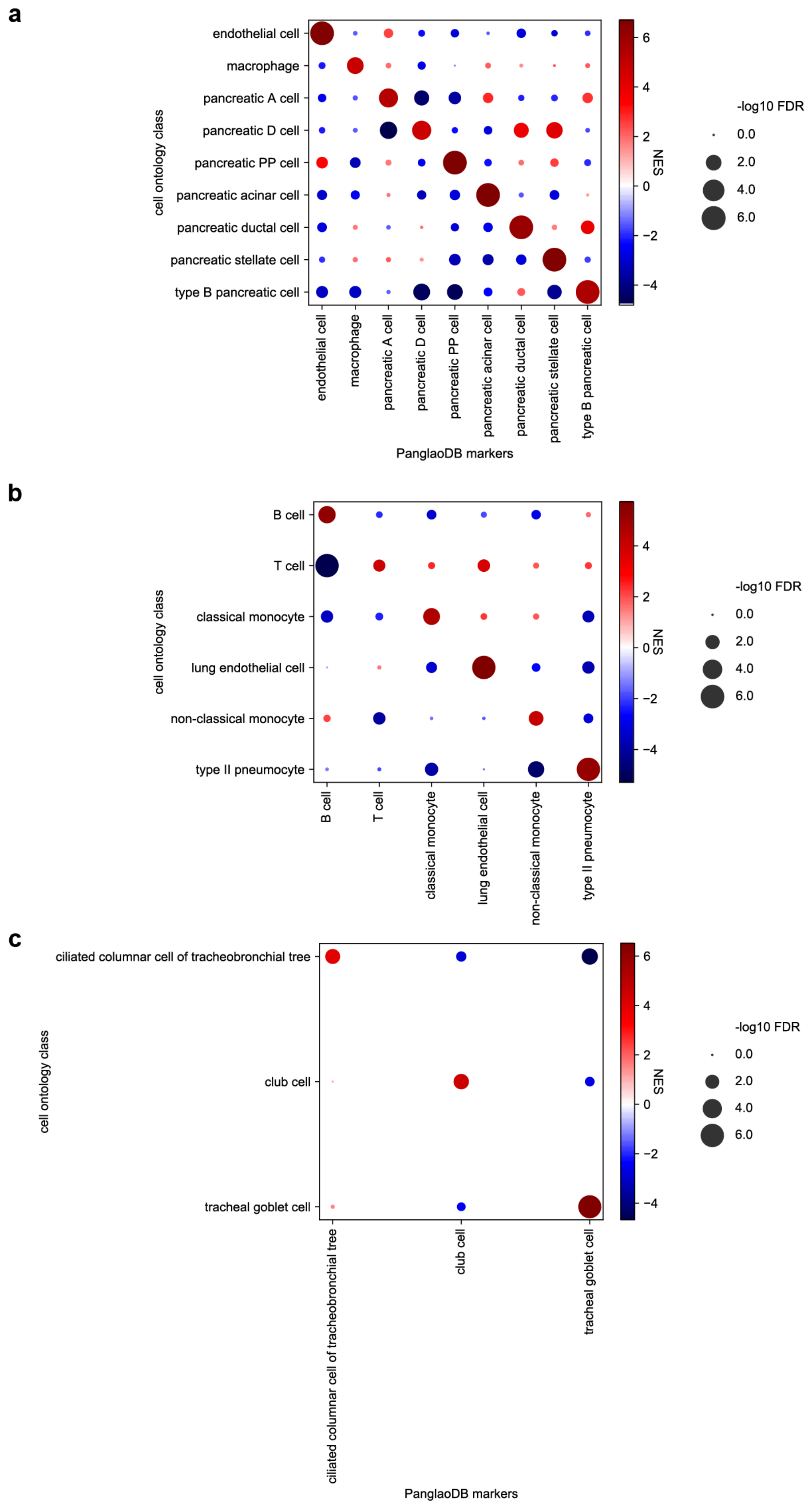

Supplementary Figure 10 Enrichment of known cell type markers among most important genes learned by Cell BLAST models. Shown are results obtained on (**a**) pancreatic references, (**b**) lung reference, and (**c**) tracheal reference in **Supplementary Table 3**. Each row corresponds to the gene importance ranking for a particular cell type learned by Cell BLAST models. Each column corresponds to manually curated markers of a particular cell type in the PanglaoDB database. NES and FDR are normalized enrichment score and false discovery rate of GSEA[41], respectively.

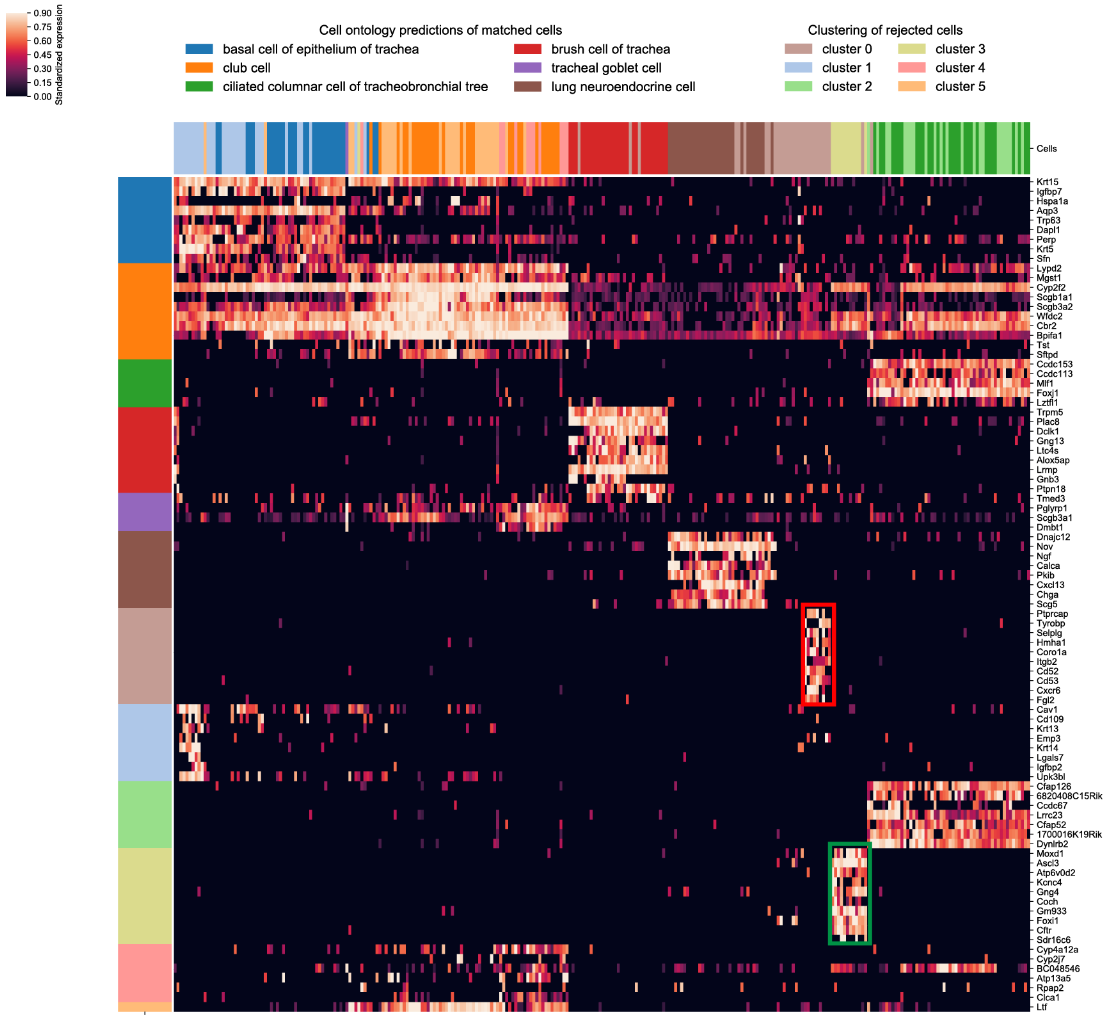

Supplementary Figure 11 Marker gene expression in Cell BLAST rejected cells compared to matched cells for the tracheal query. Rows are genes, ordered by predicted cell types / clusters of rejected cells in which they are highly expressed. Columns are cells, ordered by hierarchical clustering. Raw UMI counts are cell-wise normalized, log transformed and gene-wise standardized by scaling the highest value to 1. Ionocytes are marked by green frames, and the potentially immune-related cells are marked by red frames.

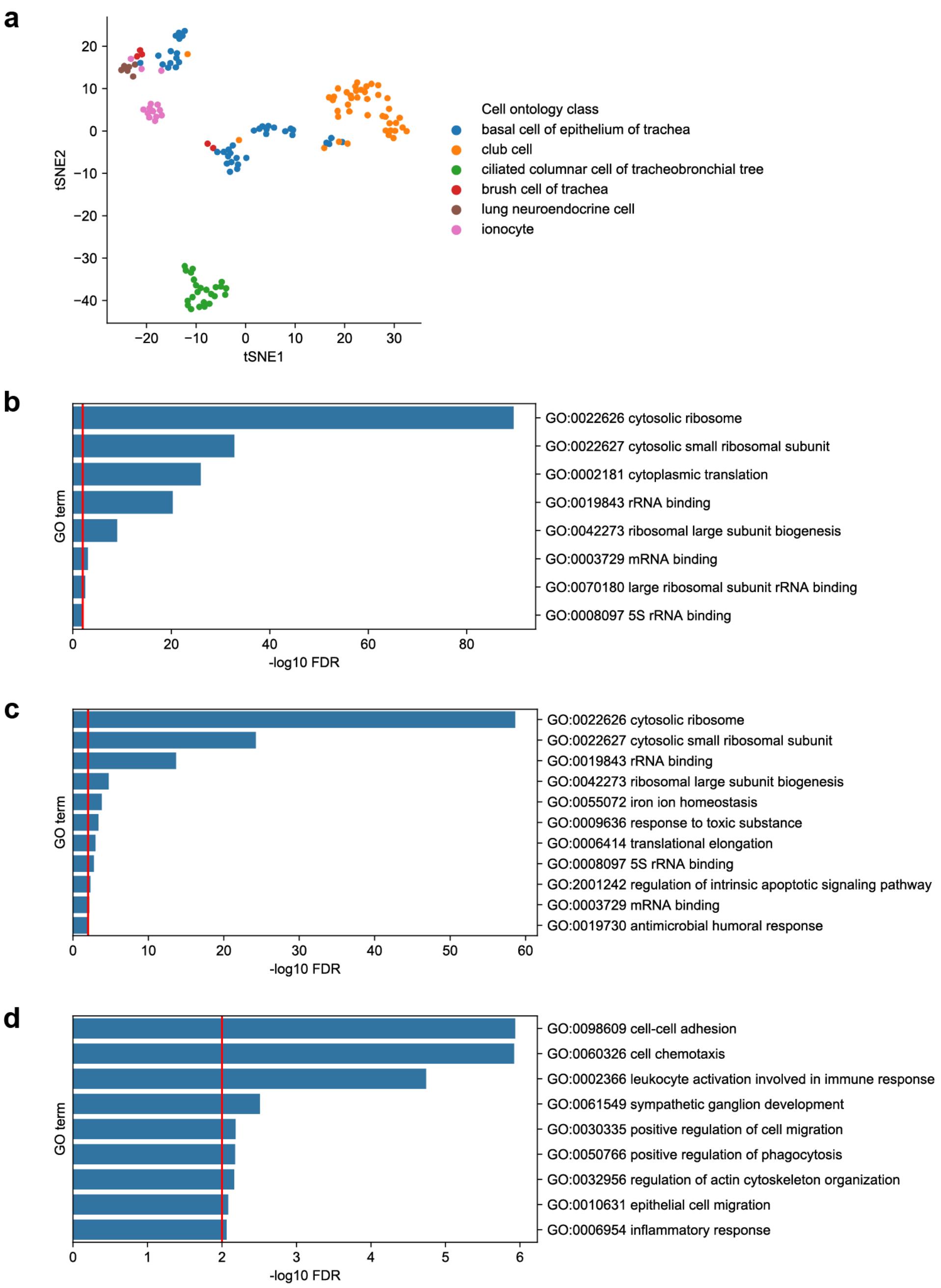

Supplementary Figure 12 Differential expression in Cell BLAST rejected cells for the tracheal query. (**a**) t-SNE visualization of Cell BLAST-rejected cells, colored by original annotation.
(**b**) GO enrichment in significantly down-regulated genes in rejected cluster 1 compared to basal cells.
(**c**) GO enrichment in significantly down-regulated genes in rejected cluster 5 compared to club cells.
(**d**) GO enrichment in significantly up-regulated genes in rejected cluster 0 compared to all other cells.
Statistical significance in (**b-d**) is evaluated by one-sided hypergeometric test. FDRs derived by the Banjamini-Hochberg procedure[42] were used to account for multiple tests. Red vertical lines indicate significance cutoff of FDR = 0.01.

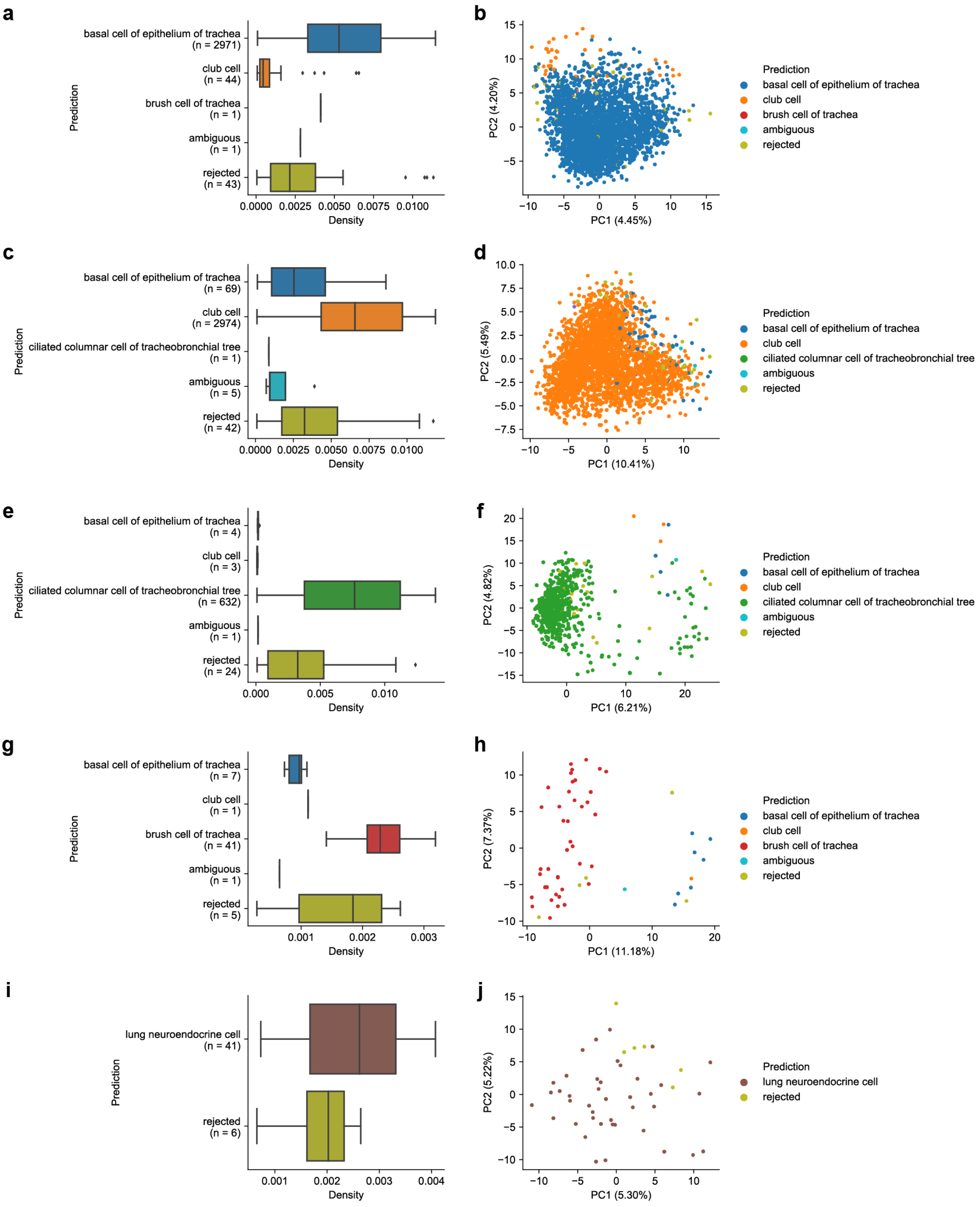

Supplementary Figure 13 PCA visualization of each cell type in the "Plasschaert" dataset, with cells colored by Cell BLAST predictions. Figures in the right column (**b, d, f, h, j**) are visualizations based on the first two principal components (PC), while figures in the left column (**a, c, e, g, i**) are Gaussian KDE (kernel density estimation) of cells grouped by prediction. Each row corresponds to cells of an originally annotated cell type, with (**a, b**) being basal cells, (**c, d**) club cells, (**e, f**) ciliated cells, (**g, h**) brush cells, (**i, j**) neuroendocrine cells. Box plots indicate the median (center lines), 1st and 3rd quartile (hinges), minimal and maximal point within 1.5 times the interquartile range starting from the hinges (whiskers).

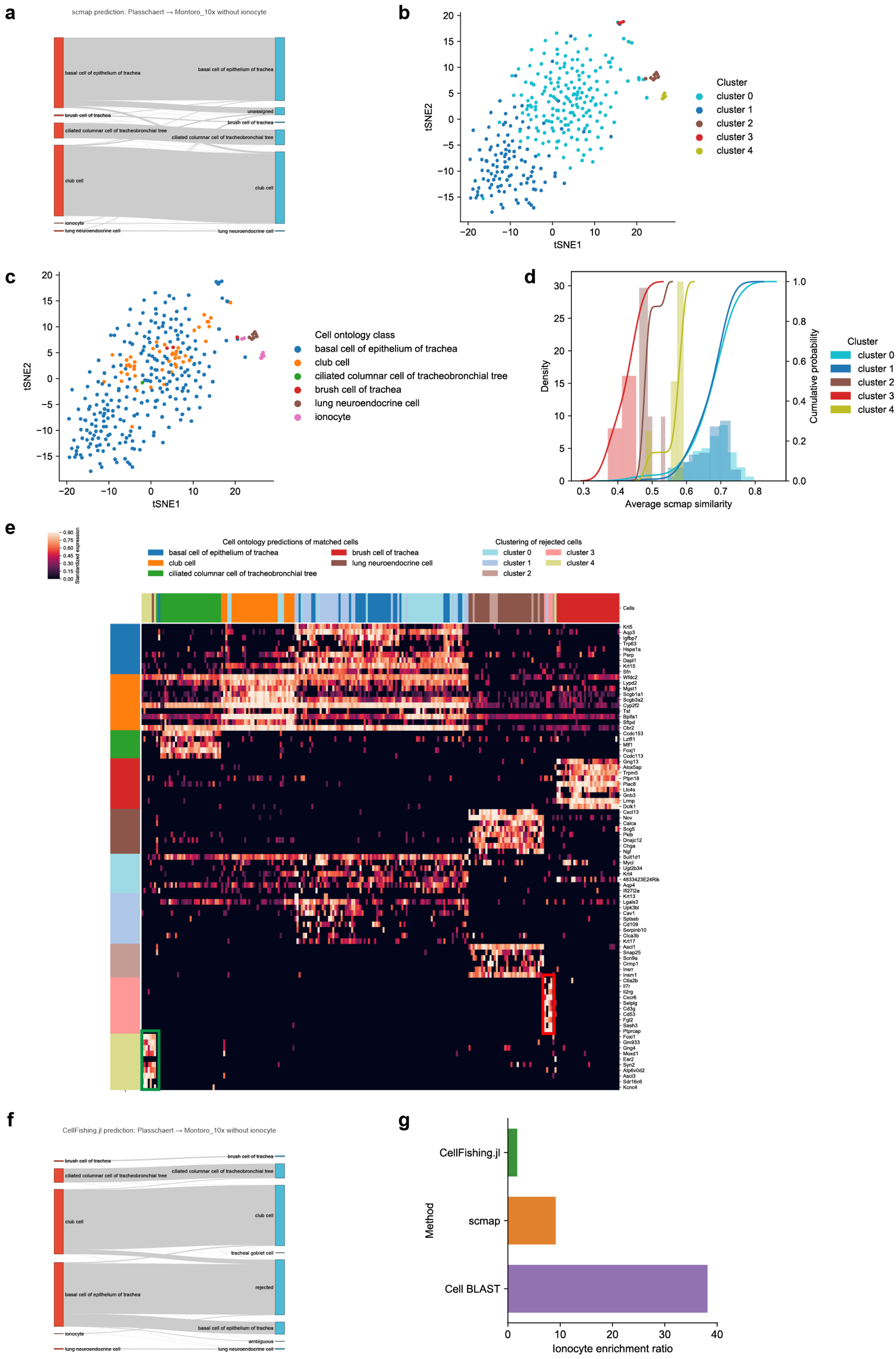
a
scmap prediction: Plasschaert → Montoro_10x without ionocyte
basal cell of epithelium of trachea
brush cell of trachea
ciliated columnar cell of tracheobronchial tree
club cell
ionocyte
lung neuroendocrine cell
unassigned
b
tSNE1
tSNE2
Cluster
cluster 0
cluster 1
cluster 2
cluster 3
cluster 4
c
Cell ontology class
basal cell of epithelium of trachea
club cell
ciliated columnar cell of tracheobronchial tree
brush cell of trachea
lung neuroendocrine cell
ionocyte
d
Density
Cumulative probability
Average scmap similarity
e
Standardized expression
Cell ontology predictions of matched cells
Clustering of rejected cells
Cells
Krt5
Aqp3
Igfbp7
Trp63
Hspa1a
Perp
Dapl1
Krt15
Sfn
Wfdc2
Lypd2
Mgst1
Scgb1a1
Scgb3a2
Cyp2f2
Tst
Bpifa1
Sftpd
Cbr2
Ccdc153
Lztfl1
Mlf1
Foxj1
Ccdc113
Gng13
Alox5ap
Trpm5
Ptpn18
Plac8
Ltc4s
Gnb3
Lrmp
Dclk1
Cxcl13
Nov
Calca
Scg5
Pkib
Dnajc12
Chga
Ngf
Sult1d1
Mycl
Ugt2b34
Krt4
4833423E24Rik
Aqp4
Ifi27l2a
Krt13
Lgals3
Upk3bl
Cav1
Sptssb
Cd109
Serpinb10
Clca3b
Krt17
Ascl1
Snap25
Scn9a
Crmp1
Insrr
Insm1
Ctla2b
Il7r
Il2rg
Cxcr6
Selplg
Cd3g
Cd53
Fgl2
Sash3
Ptprcap
Foxi1
Gm933
Gng4
Moxd1
Ear2
Syn2
Atp6v0d2
Ascl3
Sdr16c6
Kcnc4
f
CellFishing.jl prediction: Plasschaert → Montoro_10x without ionocyte
brush cell of trachea
ciliated columnar cell of tracheobronchial tree
club cell
tracheal goblet cell
basal cell of epithelium of trachea
rejected
ionocyte
ambiguous
lung neuroendocrine cell
g
CellFishing.jl
scmap
Cell BLAST
Method
Ionocyte enrichment ratio

Supplementary Figure 14 Performance of scmap and CellFishing.jl for the tracheal query. (**a**) Sankey plot comparing scmap predictions and original cell type annotations for the "Plasschaert" dataset. (**b, c**) t-SNE visualization of scmap-rejected cells, colored by unsupervised clustering (**b**) and original annotation (**c**). (**d**) Distribution of scmap similarity score for each cluster in (**b**). (**e**) Marker gene expression in scmap rejected cells compared to matched cells. Rows are genes, ordered by predicted cell types / clusters of rejected cells in which they are highly expressed. Columns are cells, ordered by hierarchical clustering. Raw UMI counts are cell-wise normalized, log transformed and gene-wise standardized by scaling the highest value to 1. Ionocytes are marked by green frames, and the potentially immune-related cells are marked by red frames. (**f**) Sankey plot comparing CellFishing.jl predictions and original cell type annotations for the "Plasschaert" dataset. (**g**) Ionocyte enrichment ratio in rejected cells for the "Plasschaert" dataset.

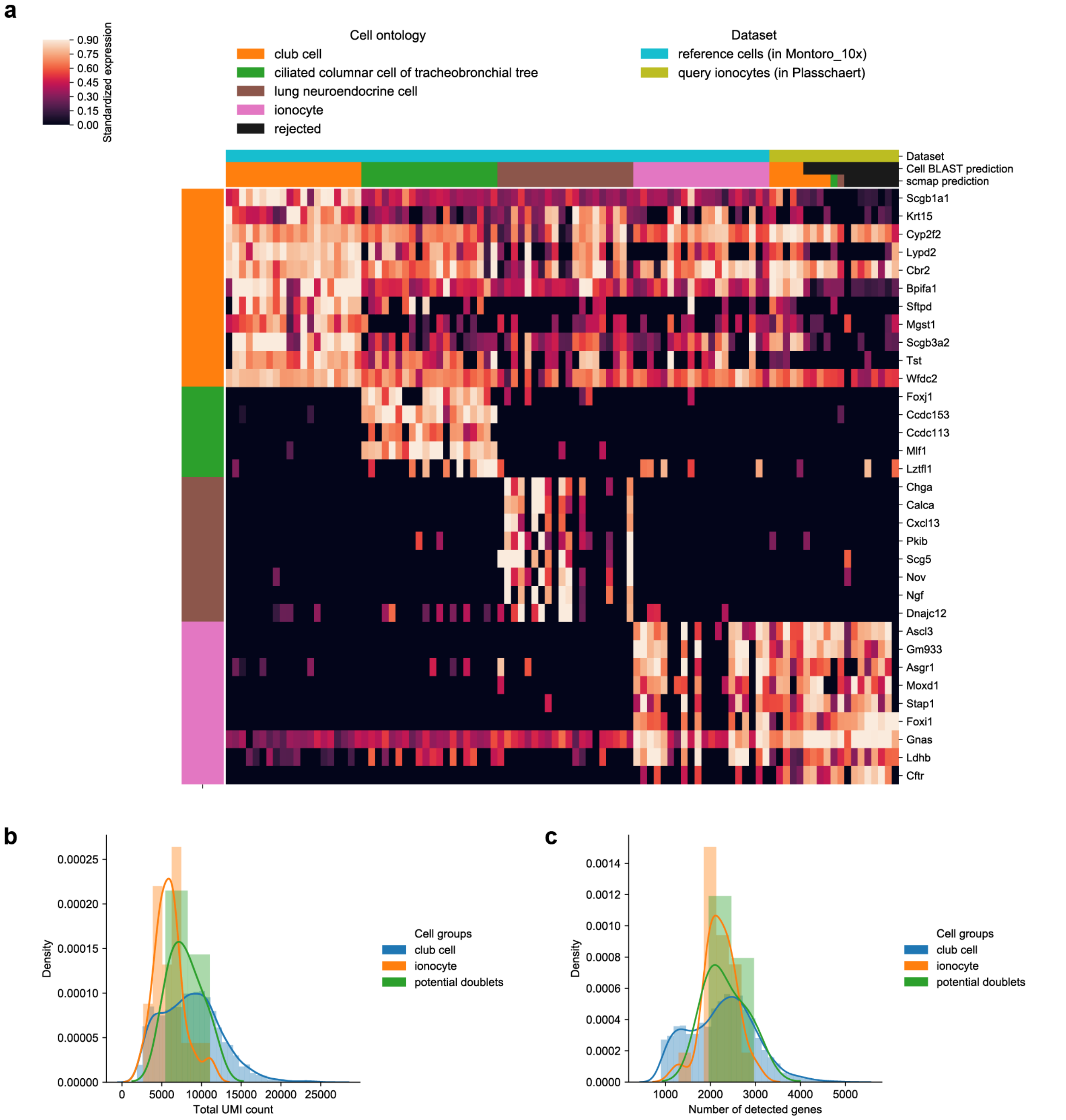

Supplementary Figure 15 Detailed examination of Cell BLAST and scmap prediction on query ionocytes. (**a**) Marker gene expression in query ("Plasschaert") ionocytes, compared to relevant reference cells. Rows are genes, ordered by cell types in which they are highly expressed. Columns are cells, ordered by cell types / predictions. The column annotation colors (top bar) have different meanings for reference cells (left section) and query ionocytes (right section). For reference cells, the colors indicate their author-annotated cell types, while for query ionocytes, the colors indicate cell type predictions made by Cell BLAST and scmap respectively. Raw UMI counts are cell-wise normalized, log transformed and gene-wise standardized by scaling the largest value to 1. (**b**) Distribution of total UMI count for potential club-ionocyte doublets compared to club cells and ionocytes. (**c**) Distribution of detected gene numbers for potential club-ionocyte doublets compared to club cells and ionocytes.

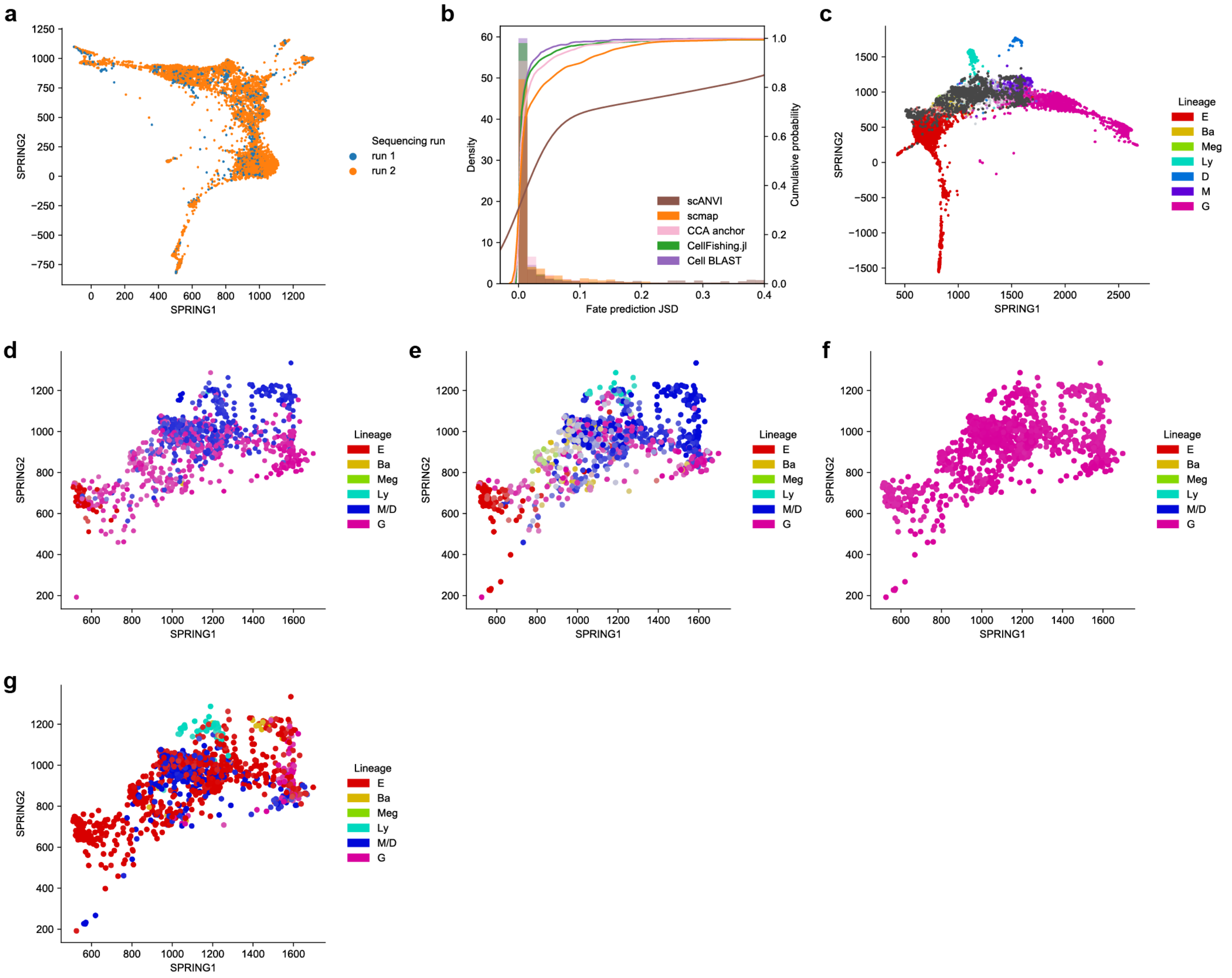

Supplementary Figure 16 Predicting continuous cell fate probabilities for hematopoietic progenitors. (**a**) SPRING visualization of the “Tusi” dataset, colored by sequencing run. (**b**) Distribution of Jensen-Shannon divergence between predicted cell fate distributions and author-provided ground truth on the “Tusi” dataset. (**c**) SPRING visualization showing the “Velten” dataset (black) aligned onto the “Tusi” dataset (colored by cell fate probabilities). (**d**-**f**) SPRING visualization of the “Velten” dataset, colored by cell fate probabilities predicted by scmap (**d**), CellFishing.jl (**e**), CCA anchor (**f**), and scANVI (**g**).

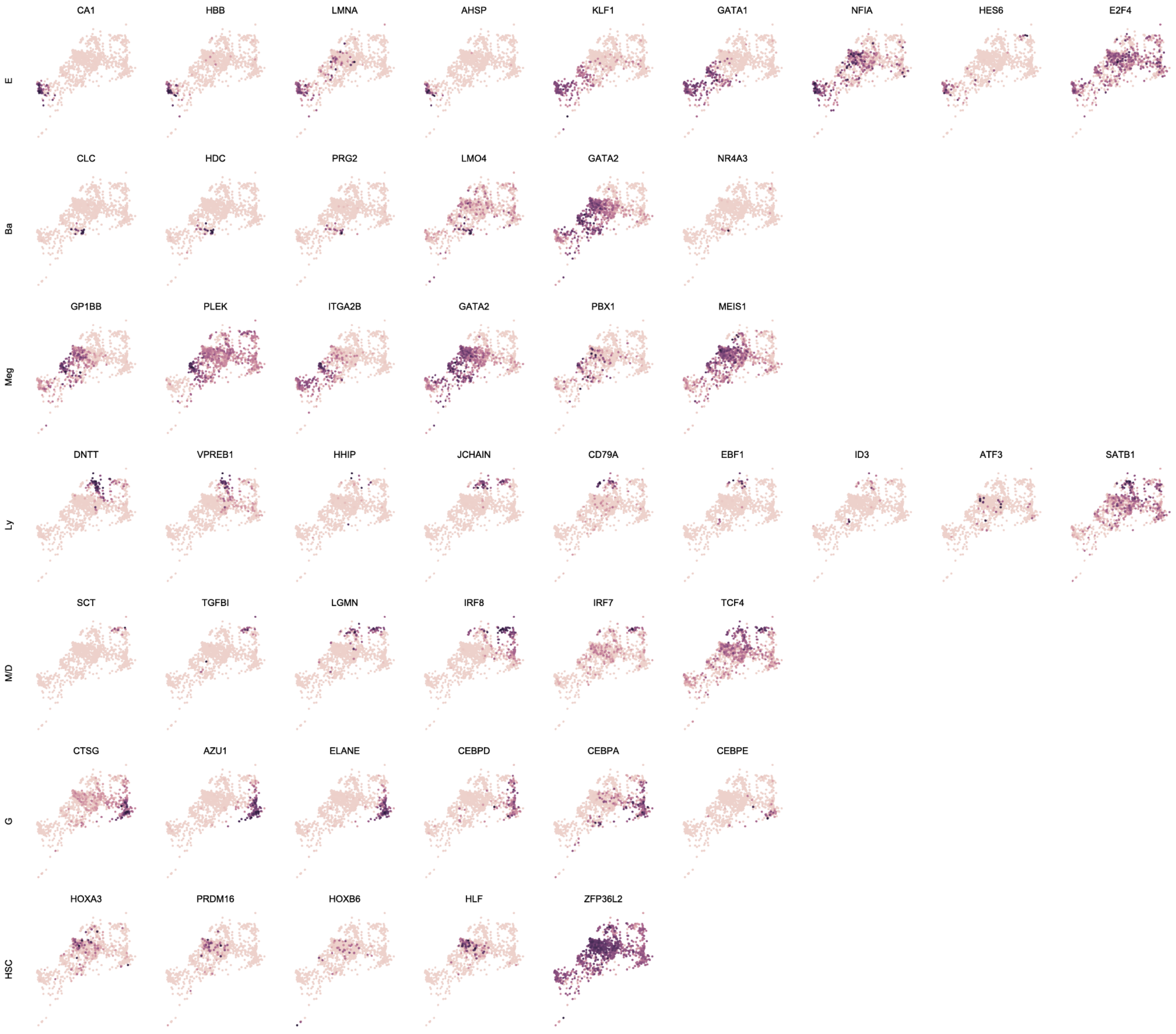

Supplementary Figure 17 Expression of known lineage markers in the "Velten" dataset.

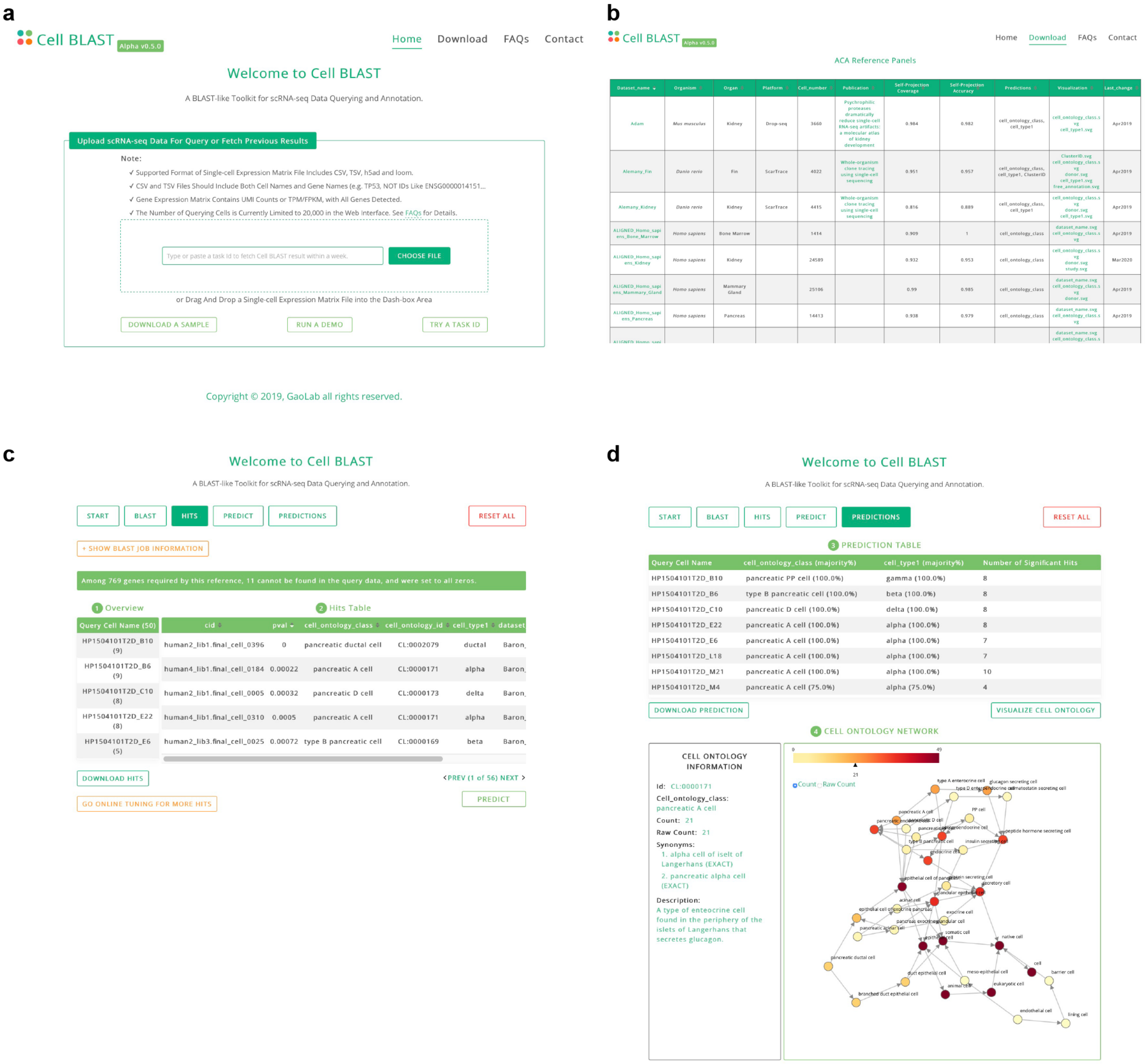

Supplementary Figure 18 ACA database and Cell BLAST Web portal. (**a**) Home page of the Cell BLAST Web interface. (**b**) List of ACA reference panels available in our Web interface (only the first dozen are shown). (**c**) Web interface showing hits of a sample query. (**d**) Web interface showing predictions for a sample query, as well as cell ontology visualization.

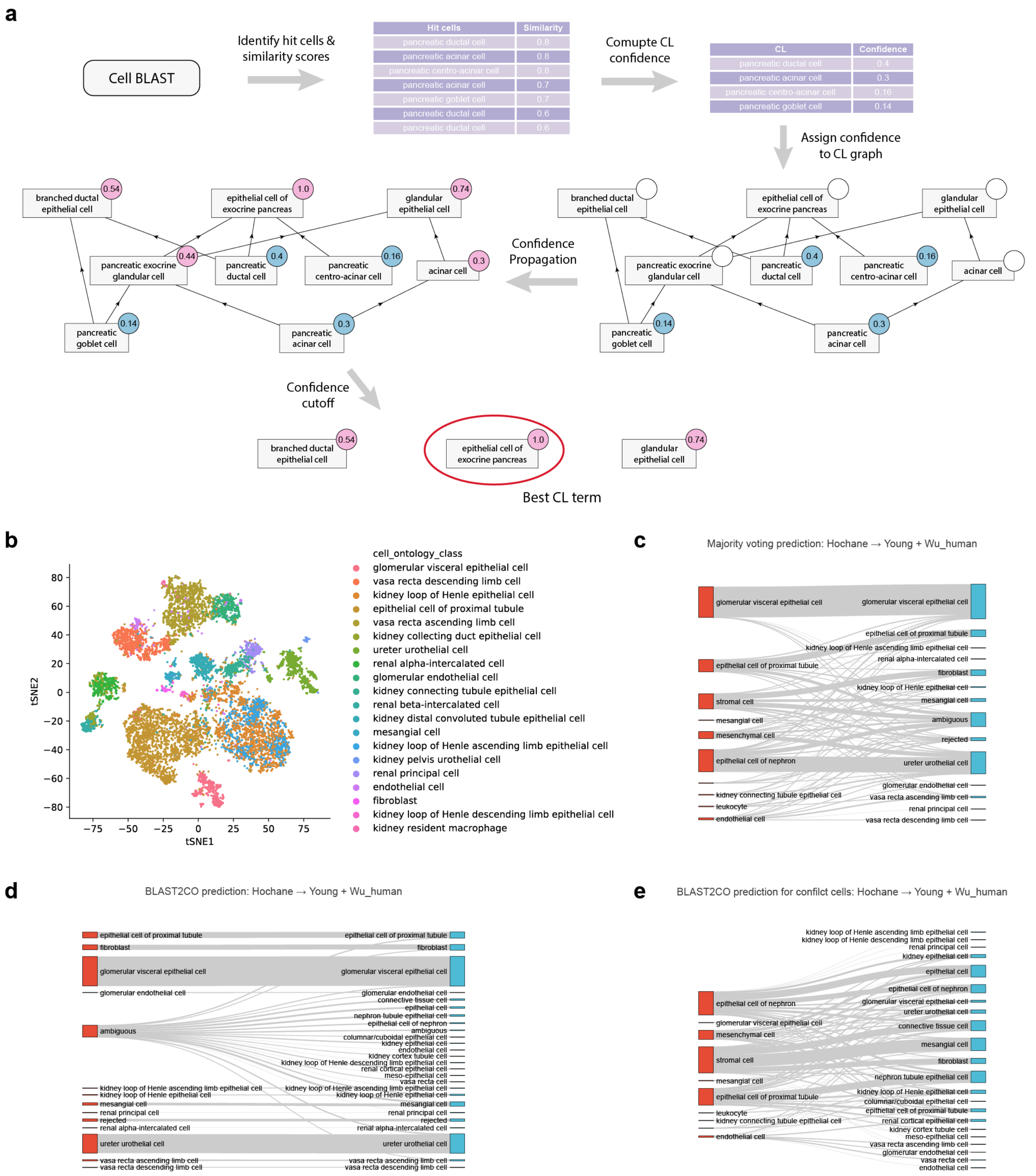

Supplementary Figure 19 BLAST2CO application with two human kidney datasets ("Young"[43] and "Wu_human"[37]) as composite reference and human fetal kidney dataset ("Hochane"[44]) as query. (**a**) Schematic diagram of the BLAST2CO workflow. (**b**) t-SNE visualization of cell embeddings for the "Young" and "Wu_human" datasets aligned together, colored by cell ontology annotation. (**c**) Sankey plot comparing majority voting predictions and original cell ontology annotations for the "Hochane" dataset. Majority voting labeled 2,205 cells as "ambiguous". (**d**) Sankey plot comparing BLAST2CO predictions and majority voting predictions for the "Hochane" dataset. Most of the majority voting "ambiguous" cells could be properly predicted by BLAST2CO, while unambiguous predictions remained the same. Overall, BLAST2CO achieved an ontology-aware CL MBA of 0.226 for these originally ambiguous results, while majority voting was 0. (**e**) Sankey plot comparing BLAST2CO predictions and original cell ontology annotations for cells where BLAST2CO prediction differs from majority voting.

References

1. Baglama J, Reichel L, Lewis BW. irlba: Fast Truncated Singular Value Decomposition and Principal Components Analysis for Large Dense and Sparse Matrices. https://cran.r-project.org/package=irlba (2019).

2. Pierson E, Yau C. ZIFA: Dimensionality reduction for zero-inflated single-cell gene expression analysis. *Genome Biol* **16**, 241 (2015).

3. Risso D, Perraudeau F, Gribkova S, Dudoit S, Vert JP. A general and flexible method for signal extraction from single-cell RNA-seq data. *Nat Commun* **9**, 284 (2018).

4. Rashid S, Shah S, Bar-Joseph Z, Pandya R. Dhaka: Variational Autoencoder for Unmasking Tumor Heterogeneity from Single Cell Genomic Data. *Bioinformatics*, (2019).

5. Eraslan G, Simon LM, Mircea M, Mueller NS, Theis FJ. Single-cell RNA-seq denoising using a deep count autoencoder. *Nat Commun* **10**, 390 (2019).

6. Maaten Lvd, Hinton G. Visualizing data using t-SNE. *Journal of machine learning research* **9**, 2579-2605 (2008).

7. McInnes L, Healy J, Melville J. UMAP: Uniform Manifold Approximation and Projection for Dimension Reduction. Preprint at https://arxiv.org/abs/1802.03426 (2018).

8. Lopez R, Regier J, Cole MB, Jordan MI, Yosef N. Deep generative modeling for single-cell transcriptomics. *Nat Methods* **15**, 1053-1058 (2018).

9. Amodio M*, et al.* Exploring single-cell data with deep multitasking neural networks. *Nat Methods* **16**, 1139-1145 (2019).

10. Ding J, Regev A. Deep generative model embedding of single-cell RNA-Seq profiles on hyperspheres and hyperbolic spaces. Preprint at https://www.biorxiv.org/content/10.1101/853457v1 (2019).

11. Deng Y, Bao F, Dai Q, Wu LF, Altschuler SJ. Scalable analysis of cell-type composition from single-cell transcriptomics using deep recurrent learning. *Nat Methods* **16**, 311-314 (2019).

12. Johnson WE, Li C, Rabinovic A. Adjusting batch effects in microarray expression data using empirical Bayes methods. *Biostatistics* **8**, 118-127 (2007).

13. Haghverdi L, Lun ATL, Morgan MD, Marioni JC. Batch effects in single-cell RNA-sequencing data are corrected by matching mutual nearest neighbors. *Nat Biotechnol* **36**, 421-427 (2018).

14. Butler A, Hoffman P, Smibert P, Papalexi E, Satija R. Integrating single-cell transcriptomic data across different conditions, technologies, and species. *Nat Biotechnol* **36**, 411-420 (2018).

15. Stuart T*, et al.* Comprehensive Integration of Single-Cell Data. *Cell* **177**, 1888-1902 e1821 (2019).

16. Korsunsky I*, et al.* Fast, sensitive and accurate integration of single-cell data with Harmony. *Nat Methods* **16**, 1289-1296 (2019).

17. Kiselev VY, Yiu A, Hemberg M. scmap: projection of single-cell RNA-seq data across data sets. *Nat Methods* **15**, 359-362 (2018).

18. Sato K, Tsuyuzaki K, Shimizu K, Nikaido I. CellFishing.jl: an ultrafast and scalable cell search method for single-cell RNA sequencing. *Genome Biol* **20**, 31 (2019).

19. Guo J*, et al.* The adult human testis transcriptional cell atlas. *Cell Res* **28**, 1141-1157 (2018).

20. Zheng GX*, et al.* Massively parallel digital transcriptional profiling of single cells. *Nat Commun* **8**, 14049 (2017).

21. Muraro MJ*, et al.* A Single-Cell Transcriptome Atlas of the Human Pancreas. *Cell Syst* **3**, 385-394 e383 (2016).

22. Hashimshony T*, et al.* CEL-Seq2: sensitive highly-multiplexed single-cell RNA-Seq. *Genome Biol* **17**, 77 (2016).

23. Xin Y*, et al.* RNA Sequencing of Single Human Islet Cells Reveals Type 2 Diabetes Genes. *Cell Metab* **24**, 608-615 (2016).

24. Verboom K*, et al.* SMARTer single cell total RNA sequencing. *Nucleic Acids Res* **47**, e93 (2019).

25. Lawlor N*, et al.* Single-cell transcriptomes identify human islet cell signatures and reveal cell-type-specific expression changes in type 2 diabetes. *Genome Res* **27**, 208-222 (2017).

26. Segerstolpe A*, et al.* Single-Cell Transcriptome Profiling of Human Pancreatic Islets in Health and Type 2 Diabetes. *Cell Metab* **24**, 593-607 (2016).

27. Picelli S, Faridani OR, Bjorklund AK, Winberg G, Sagasser S, Sandberg R. Full-length RNA-seq from single cells using Smart-seq2. *Nat Protoc* **9**, 171-181 (2014).

28. Enge M*, et al.* Single-Cell Analysis of Human Pancreas Reveals Transcriptional Signatures of Aging and Somatic Mutation Patterns. *Cell* **171**, 321-330 e314 (2017).

29. Baron M*, et al.* A Single-Cell Transcriptomic Map of the Human and Mouse Pancreas Reveals Inter- and Intra-cell Population Structure. *Cell Syst* **3**, 346-360 e344 (2016).

30. Klein AM*, et al.* Droplet barcoding for single-cell transcriptomics applied to embryonic stem cells. *Cell* **161**, 1187-1201 (2015).

31. Adam M, Potter AS, Potter SS. Psychrophilic proteases dramatically reduce single-cell RNA-seq artifacts: a molecular atlas of kidney development. *Development* **144**, 3625-3632 (2017).

32. Macosko EZ*, et al.* Highly Parallel Genome-wide Expression Profiling of Individual Cells Using Nanoliter Droplets. *Cell* **161**, 1202-1214 (2015).

33. Plasschaert LW*, et al.* A single-cell atlas of the airway epithelium reveals the CFTR-rich pulmonary ionocyte. *Nature* **560**, 377-381 (2018).

34. Montoro DT*, et al.* A revised airway epithelial hierarchy includes CFTR-expressing ionocytes. *Nature* **560**, 319-324 (2018).

35. Bach K*, et al.* Differentiation dynamics of mammary epithelial cells revealed by single-cell RNA sequencing. *Nat Commun* **8**, 2128 (2017).

36. Tabula Muris C*, et al.* Single-cell transcriptomics of 20 mouse organs creates a Tabula Muris. *Nature* **562**, 367-372 (2018).

37. Wu H, Uchimura K, Donnelly EL, Kirita Y, Morris SA, Humphreys BD. Comparative Analysis and Refinement of Human PSC-Derived Kidney Organoid Differentiation with Single-Cell Transcriptomics. *Cell Stem Cell* **23**, 869-881 e868 (2018).

38. Philippeos C*, et al.* Spatial and Single-Cell Transcriptional Profiling Identifies Functionally Distinct Human Dermal Fibroblast Subpopulations. *J Invest Dermatol* **138**, 811-825 (2018).

39. Park J*, et al.* Single-cell transcriptomics of the mouse kidney reveals potential cellular targets of kidney disease. *Science* **360**, 758-763 (2018).

40. Giraddi RR*, et al.* Single-Cell Transcriptomes Distinguish Stem Cell State Changes and Lineage Specification Programs in Early Mammary Gland Development. *Cell Rep* **24**, 1653-1666 e1657 (2018).

41. Subramanian A*, et al.* Gene set enrichment analysis: a knowledge-based approach for interpreting genome-wide expression profiles. *Proc Natl Acad Sci U S A* **102**, 15545-15550 (2005).

42. Hochberg Y, Benjamini Y. More powerful procedures for multiple significance testing. *Stat Med* **9**, 811-818 (1990).

43. Young MD*, et al.* Single-cell transcriptomes from human kidneys reveal the cellular identity of renal tumors. *Science* **361**, 594-599 (2018).

44. Hochane M*, et al.* Single-cell transcriptomics reveals gene expression dynamics of human fetal kidney development. *PLoS Biol* **17**, e3000152 (2019).
