# Supplementary material for: Searching large-scale scRNA-seq databases via unbiased cell embedding with Cell BLAST
Source: Nat Commun. 2020 Jul 10;11:3458. doi: 10.1038/s41467-020-17281-7 (PMC7351785; doi:10.1038/s41467-020-17281-7)
Supplement: Supplementary file 8 — Description of Additional Supplementary Files [file 41467_2020_17281_MOESM8_ESM.pdf]

Title: Supplementary Data 1
Description: Cell type composition in datasets used for batch effect correction benchmarking.

Title: Supplementary Data 2
Description: Detailed information for datasets in ACA.

Title: Supplementary Data 3
Description: Expected prediction matrices for query-based cell typing.

Title: Supplementary Data 4
Description: Source data for all benchmarking experiments.

Title: Supplementary Software
Description: The Cell BLAST Python package.
